# Human Mesenchymal Stem Cell Combined with a New Strontium-Enriched Bioactive Glass: An *ex-vivo* Model for Bone Regeneration

**DOI:** 10.3390/ma12213633

**Published:** 2019-11-05

**Authors:** Devis Bellucci, Elena Veronesi, Valentina Strusi, Tiziana Petrachi, Alba Murgia, Ilenia Mastrolia, Massimo Dominici, Valeria Cannillo

**Affiliations:** 1 Department of Engineering “Enzo Ferrari”, University of Modena and Reggio Emilia, Via P. Vivarelli 10, 41125 Modena, Italy; devis.bellucci@unimore.it; 2 Department of Medical and Surgical Sciences for Children & Adults, University of Modena and Reggio Emilia, Hospital of Modena, Via del Pozzo 71, 44125 Modena, Italy; elena.veronesi@unimore.it (E.V.); tiziana.petrachi@unimore.it (T.P.); alba.murgia@unimore.it (A.M.); ilenia.mastrolia@unimore.it (I.M.); massimo.dominici@unimore.it (M.D.); 3 Scientific and Technological Park of Medicine “Mario Veronesi”, via 29 Maggio 6, 41037 Mirandola, Italy; valentina.strusi@tpm.bio

**Keywords:** bioactive glass, mesenchymal stem cells, bone regeneration, cell culture, strontium, magnesium

## Abstract

A 3D cellular model that mimics the potential clinical application of a biomaterial is here applied for the first time to a bioactive glass, in order to assess its biological potential. A recently developed bioactive glass (BGMS10), whose composition contained strontium and magnesium, was produced in the form of granules and fully investigated in terms of biocompatibility in vitro. Apart from standard biological characterization (Simulated Body Fluid (SBF) testing and biocompatibility as per ISO10993), human bone marrow mesenchymal stromal/stem cells (BM-MSCs) were used to investigate the performance of the bioactive glass granules in an innovative 3D cellular model. The results showed that BGMS10 supported human BM-MSCs adhesion, colonization, and bone differentiation. Thus, bioactive glass granules seem to drive osteogenic differentiation and thus look particularly promising for orthopedic applications, bone tissue engineering and regenerative medicine.

## 1. Introduction

A fundamental clinical challenge in dentistry, orthopedics, and reconstructive surgery is the treatment of bone defects and lacunae, caused by trauma or disease. In this context, in the last decades calcium phosphates, such as hydroxyapatite, and bioactive glasses have been widely employed as synthetic bone grafts by virtue of their excellent biocompatibility and their ability of direct bonding to bone, i.e., their so-called bioactivity [1,2,3,4,5].

Recently, 45S5 Bioglass^®^ (45S5) is the most used glass for biomedical applications, although some drawbacks of this material have emerged over time, such as its tendency to crystallize upon thermal treatments required to produce specific products, i.e., glass coatings, porous scaffolds for bone tissue engineering, and composites based on glass and calcium phosphates. In fact, crystallization: (1) makes difficult, or even inhibits, the sintering process which is necessary to obtain fully dense samples of specific shape, starting from glass powders (or from a proper mixture of glass and calcium phosphate powders); (2) retards the reactivity in vivo of the final product; and (3) may cause cracks in the implant or implant’s instability in vivo, since the crystalline phases which formed during sintering are less soluble in physiological environment than the residual glassy phase, which is typically dissolved preferentially [4,6,7,8]. Moreover, it should be stressed that the regenerative potential of bioglasses and bioceramics is strongly influenced by their ionic dissolution products [9], which may be released too slowly by partly crystallized samples. In recent years, the important biological effects of specific ions not present in the original 45S5’s composition (24.4 *mol* % Na_2_O, 26.9 *mol*% CaO, 46.1 *mol*%, SiO_2,_ and *2.6 mol*% P_2_O_5_), such as zinc, copper, magnesium, boron, fluorine, and strontium, have been highlighted by several authors [9,10,11,12,13]. These ions are able to provide antibacterial properties, to stimulate angiogenesis, neovascularization, and osteogenesis. Therefore, therapeutic metallic elements (Ag^+^, Sr^2+^, Cu^2+^, etc.) have been incorporated into the compositions of novel bioactive glasses, with the aim to promote a specific and more advanced cell response [9] and/or to enhance the thermal stability of a given bioglass composition [14]. In particular, strontium (Sr) and magnesium (Mg) seem to play a relevant role in bone formation and remodeling. Sr may be used to enhance osteogenesis and reduce bone resorption, since such element has been shown to simultaneously inhibit bone resorption from osteoclasts and stimulate bone formation by osteoblasts. For this reason, Sr is currently used as strontium ranelate in the treatment of osteoporosis, an important bone disease that affects hundreds of millions of people worldwide [15,16,17]. Several investigations regarding bioglasses where strontium partially substitutes calcium have demonstrated its beneficial effect in stimulating bone remodeling [18,19]. On the other hand, magnesium is a very interesting ion as a component of bioglasses by virtue of its fundamental role in bone tissue development and in several biological processes in the human body, in addition to its common use to modify and improve thermal, mechanical, and physical performance of bioactive silicate glasses [12]. 

Based on these considerations, recently, the new BGMS10 bioactive glass (composition: 2.3 mol % Na_2_O; 2.3 mol % K_2_O; 25.6 mol % CaO; 10.0 mol % MgO; 10.0 mol % SrO; 2.6 mol % P_2_O_5_; and 47.2 mol % SiO_2_) has been developed [14]. BGMS10 is characterized by both an excellent in vitro bioactivity, which has been evaluated in a Simulated Body Fluid solution (SBF) according to the protocol by Kokubo et al. [20], and an ultra-high crystallization temperature (T_C_ = 932 °C [14]), which makes such glass an ideal candidate whenever a thermal treatment is required to produce a specific final product. For comparison, it should be noted that 45S5 has a crystallization temperature as low as 650–690 °C [7]. 

In this work, for the first time, BGMS10 has been produced in form of granules and tested in terms of biological performance in vitro, in particular with respect to human mesenchymal stem cells. First of all, biocompatibility was assessed as recommended by international standard procedures (ISO 10993), with the aim of excluding cytotoxicity effects of the bioactive glass itself. Subsequently, the osteoinductivity of BGMS10 was evaluated, by using a cellular model that mimicked the potential clinical application of the biomaterial itself in the orthopedic field. In fact, bone tissue undergoes a life-long process of bone remodeling, where osteoclasts break bone down and osteoblasts make new bone tissue [21]. Osteoblasts as derived from a differentiation process of human bone marrow mesenchymal stromal/stem cells (BM-MSCs), as discovered in 1968 [22], for several decades, BM-MSCs have been used for pre-clinical and clinical bone tissue engineering studies. In 2D standard culture BM-MSCs are seeded into a culture vessel, promptly adhere to the plastic surface and proliferate, once reaching the confluence, BM-MSCs are able to differentiate in bone lineage adding proper stimuli to the culture media [23]. 

Here, a 3D cellular model, using human BM-MSCs, was used to investigate the adhesion, colonization and bone differentiation ability of BM-MSC combined with BGSM10. It is worth noting that such 3D cellular model has been previously proposed and utilized to assess the osteoinductivity of other biomaterials types, such as ceramic scaffolds [24,25], but it was utilized for the first time in this work to investigate bioactive glasses.

## 2. Materials and Methods 

### 2.1. Glass Preparation and Characterization

The preparation of the BGMS10 glass by means of a classical melt-quenching route has been already reported elsewhere [13,14]. Briefly, the raw powder reagents (SiO_2_, Ca_3_(PO_4_)_2_, Na_2_CO_3_, CaCO_3_, K_2_CO_3_, SrCO_3_, Mg(OH)_2_·5H_2_O, Carlo Erba Reagenti, Rodano-Milano, Italy) were melted at 1450 °C in a Pt crucible in air. The molten bioglass has been rapidly quenched in water to obtain a frit, subsequently left to dry at 110 °C for 12 hours. Finally, the frit was ground and sieved to obtain granules with grain size between 300 and 600 μm. 

The phase composition of the granules and the possible formation of undesired crystalline phases were studied by means of X-ray diffraction (XRD: X’Pert PRO; Panalytical, Almelo, the Netherlands). Data collection was performed by a 2θ scan method in the range of 10–70° using Cu-kα X-ray line (step size: 0.017° 2θ; scan step time: 61.59 s). 

### 2.2. SBF Testing

Prior to the biological evaluation, the bioactivity of BGMS10 granules was confirmed in vitro in Simulated Body Fluid (SBF). SBF was prepared according to the Kokubo protocol [20] and stored at 37 °C. The pH was adjusted at 7.4 before use. BGMS10 granules were soaked in SBF using a 75 mg glass to 50 mL SBF ratio, as recommended in [26], and placed in an incubator at 37 °C. The samples were incubated for 1, 3, and 7 days. At the end of each time period, the granules were collected by filtration, gently rinsed with DI water and then with acetone [26]. Finally, the samples were left to dry at room temperature in the laboratory environment for 24 h. The precipitation of a hydroxyapatite layer on the surface of the BGMS10 granules was investigated by means of an environmental scanning electron microscope, ESEM (ESEM Quanta 200-FEI Company, Eindhoven, The Netherlands), coupled with an Energy-Dispersive X-ray (EDS) microanalysis system (INCA, Oxford Instruments, Abingdon, UK). The microscope was operated in a low-vacuum mode with a pressure of 0.5 Torr.

### 2.3. Biocompatibility of BGMS10 According to ISO 10993-5

Biocompatibility was investigated according to ISO10993 [27]. Eluates were prepared incubating overnight BGMS10 granules with Dulbecco Modified Eagle Medium-DMEM (Gibco) at 37 °C [28]. 

A fibroblast cell line recommended by ISO10993, namely L929, was seeded at a density of 10000 cells per 96 wells (Corning), with expansion media composed by DMEM supplemented with 10% Fetal Bovine Serum (FBS, Euroclone) and 1% Penicillin-Streptomycin (Gibco). Twenty-four hours after seeding, the culture media was substituted with eluates at different concentrations: 100%, 46.41%, 21.54%, and 10% (*v/v* in DMEM and 1% Penicillin/Streptomicin-Gibco). Negative control was set using the expansion media and a positive control was obtained with addition of 0.1% SDS to the expansion media. Then, after 24 hours, cultures were observed at the microscope Observer A1 (Zeiss) at magnification of 10X. To better quantify cytotoxicity, MTT assay was performed as well. Tetrazolium salts were added to the cell culture in a solution 1mg/ml in DMEM, and incubated for 2 hours as recommended by ISO10993 [29] and then were reduced by NAD(P)H oxidoreductase, resulting in formazan. Formazan, a purple compound, was measured with the multiplate reader Enspire (Perkin Elmer, Waltham, MA, 02451 USA). MTT assay was performed in sextuplicate.

### 2.4. Combination of BM-MSCs with BGMS10

BM-MSCs (a kindly gift from prof. Massimo Dominici), isolated from bone marrow of three healthy donors, were thawed, seeded at a density of 6000 cell/cm^2^ in 2D standard culture vessels (Greiner BIO-one). The cells were amplified for four passages, with amplification media composed by α-minimum essential medium (MEM) without nucleosides (Gibco, Invitrogen), supplemented with 8% platelet lisate, 1% L-Glutamine (Gibco Invitrogen), 1 UI/mL heparin (Sigma-Aldrich), and 1% Penicillin-Streptomycin, (Gibco). Once reached the confluence, BM-MSCs were detached using a solution of Trypsin/EDTA (Gibco) and then blocked with blocking media composed by DMEM supplemented with 10% FBS and 1% Penicillin/Streptomycin, centrifuged and re-suspended in expansion media, following the proper dilution suitable for the next experiment. 

BGMS10 granules (0.05 g), previously sterilized by UV light for 2 hours [30], were loaded in each well of a MW96 low adhesion plate (Corning) and promptly used for cell seeding without washes. A pivotal study was performed to assess the suitable cell density, reported as ratio of number of cells for mg of biomaterial: 1500/mg, 3000/mg and 6000/mg in expansion media and incubated in control condition (5% CO_2_, 37 °C). The expansion media was changed 3 times per week until the end of the experiment.

Finally, to visualize the cells adhered to the biomaterial, Crystal violet staining was performed at 1 hour, 4 days, 7 days and 14 days post seeding. Samples were fixed with ice methanol (Fisher Chemical), stained with 0.4% of Crystal violet solution (Sigma-Aldrich), washed again and left to dry. 

### 2.5. Bone Differentiation Assay on BGMS10

After selecting the proper cell density, BM-MSCs samples underwent bone differentiation assay. BM-MSCs were seeded at 3000/mg of biomaterials, i.e., BGMS10, (*n* = 6), maintained in expansion media for one week and then shifted to Osteo Differentiation media composed by expansion media supplemented with 10 nM dexamethasone, 0.1 mM L-ascorbic acid-2-phosphate, 10 mM Beta-glycerol phosphate (all from Sigma Aldrich) for 2 weeks and 100 ng/ml of BMP2 (PreProtech), added only in the second week. As proper controls, BM-MSCs were seeded at 3000/mg of biomaterials (*n* = 6) and maintained in expansion media until the end of the differentiation assay. To investigate the cell ability to differentiate in bone lineage in vitro, BM-MSCs were seeded at 10000 cell/cm^2^ in 2D standard culture and after 3 days, expansion media was substituted with Osteo Differentiation media (*n* = 3). 2D control cultures were obtained keeping BM-MSCs in expansion media (*n* = 3).

To detect mineralized tissue, Alizarin Red S staining was performed. Osteo Differentiation or expansion media were discarded, cultures were washed with water, and incubated with 2% pH 5.5 of Alizarin (Sigma Aldrich), washed again with water, PBS and left to dry.

Once dried, 2D cultures were observed with an Observer (Zeiss) at magnification of 10x and 3D cultures were observed by means of an AxioZoom V16 microscope (Zeiss, Jena, Germany) (Zeiss). The images were acquired at magnification of 100X, resolution 0.8 µm, depth of field 12 µm, and with an exposure time of 639 ms. Multiple thicknesses were imaged to show the various depth of cell growth in the 60–200 μm range. Image reconstructions were then performed by ZEN pro Software (Zeiss, Jena, Germany).

### 2.6. Statistics

All results were reported as mean ± standard deviation (mean ± SD) and Student’s *t*-test was used to assess the significant differences between specimens. 

## 3. Results and Discussion

### 3.1. BGMS10 Characterization

**Figure 1:**
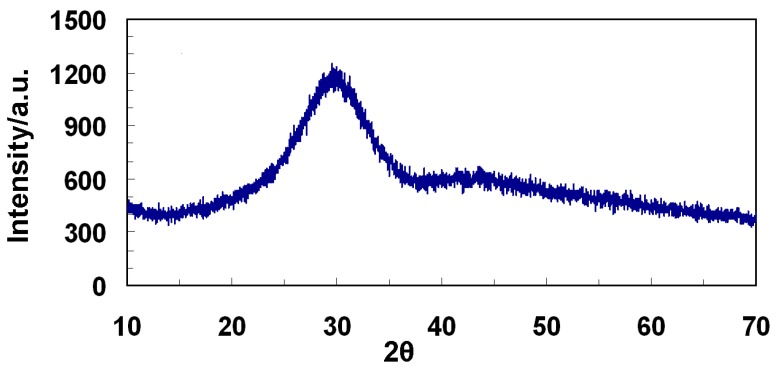
X-ray diffraction (XRD) spectrum of BGMS10 granules as prepared.

A detailed thermal characterization of BGMS10 has been already reported in a recent work devoted to sintered BGMS10 samples [14]. Compared to 45S5, the BGMS10 is characterized by a remarkably higher crystallization temperature (932 °C), larger processing window and it can be sintered at lower temperature (737 °C vs. 1000–1100 °C, which is typically reported for 45S5 [6,7]), thus maintaining its amorphous nature and potential pronounced bioactivity after the thermal treatment. Moreover, BGMS10 could result particularly interesting by virtue of its biological performance, since it contains ions, such as strontium and magnesium, whose biological relevance has been confirmed by the literature [9,12,15,16,17]. In this work, for the first time, BGMS10 has been produced in form of particulate (granules with grain size between 300 and 600 μm) to have a suitable bone substitute material for specific applications in dentistry and orthopedics, i.e., to fill and repair bone defects. It is known that the higher reactivity of bioactive glasses compared to typical bioceramics, such as hydroxyapatite, is due both to their chemical composition and their amorphous nature. Figure 1 shows the XRD spectrum acquired on the granules, which is characterized the typical trend of amorphous glasses, without any undesired crystalline peak due to possible crystallization phenomena, which may have occurred during the production of the material. 

### 3.2. SBF Testing 

**Figure 2:**
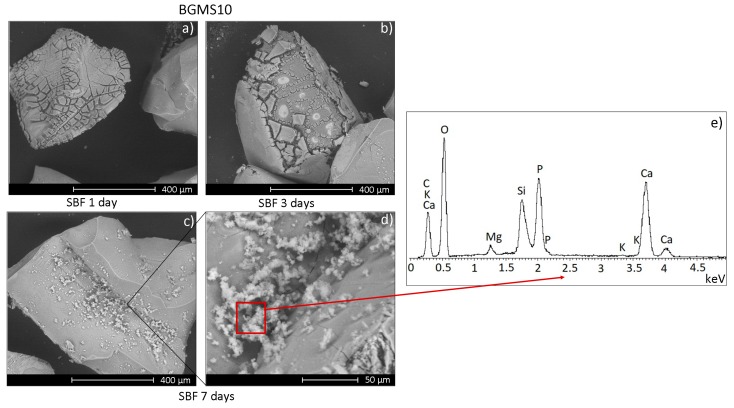
Scanning electron microscope (SEM) micrographs of BGMS10 granules after soaking in Simulated Body Fluid (SBF) for 1 day (**a**), 3 days (**b**), and 7 days (**c**,**d**); (**e**) reports the results of the an Energy-Dispersive X-ray (EDS) analysis performed on the globular precipitates shown in (**d**) and formed on the granules after 7 days in SBF.

In view of the biological characterization of BGMS10 granules, their bioactivity has been preliminary assessed in vitro in SBF, which is an acellular solution with ion concentration analogous to that of human blood plasma. Such protocol, originally developed by Kokubo et al. [20], has been re-proposed more recently and properly modified to take into account bioactive glass granules and powders [26]. Figure 2 shows the surface of BGMS10 granules after immersion in SBF for increasing times. 

After 1 day in SBF the granules’ surface is characterized by several cracks, while after 7 days it is possible to observe the formation of white globular precipitates with the typical morphology of hydroxyapatite. The results of the EDS analysis (Figure 2e) performed on the precipitates reports a Ca/P stoichiometric value analogous to that of hydroxyapatite (~1.67 [31]), apart from local fluctuations, thus confirming the bioactivity of BGMS10 granules. In fact, SBF tests aim to qualitatively estimate the bone-bonding ability of a given biomaterial by evaluating the ability of hydroxyapatite to nucleate on the sample after soaking in SBF. Such superficial layer of hydroxyapatite is similar to the biological apatite, i.e., the mineral component of bone tissue, therefore it is able to enhance the attachment of implants to bone supporting new bone growth along the implant itself. This property is known as osteoconductivity [32]. It should be stressed that the formation of hydroxyapatite on a given bioglass in SBF depends on the glass composition, production route (melting or sol-gel) and, in particular, morphology (fully dense bodies, porous scaffolds, granules, pellets…), which means very different specific surface areas exposed to the SBF or the physiological environment [26]. The cracks and fractures on the granules’ surface after 1 and 3 days in SBF (Figure 2a,b) are ascribable to the drying and subsequent delamination of a silica gel film, whose formation is among the initial steps of the process which leads to the precipitation of hydroxyapatite on bioactive glasses, according to the model originally proposed by L.L. Hench [5] and subsequently confirmed by several studies both in vitro and in vivo [33,34,35,36]. The findings here discussed, regarding the BGMS10 bioactivity, are analogous to those previously reported about bioactive glasses of different compositions containing strontium and/or magnesium, further confirming that the presence of these elements have negligible negative effects on the samples’ bioactivity [13].

### 3.3. Biocompatibility of BGSM10 According to ISO10993

Biocompatibility of BGMS10 was assessed according to ISO10993. Eluates were added to L929 and after 24 hours specimens were observed. In the positive control, cell debris was detected suggesting that cells were dead. On the contrary, L929 of negative control and tested samples resulted in viable cells, adherent with a fibroblastoid morphology, and no debris was observed. To further investigate a possible toxic event, MTT assay was performed as recommended by ISO10993, since it is a fast assay and it is commonly applied in cytotoxicity studies. Positive control showed an average of 8.15% ± 0.02%, significantly different from the negative control that has been normalized at 100.00% ± 0.08% (*p-*value < 0.05). 

**Figure 3:**
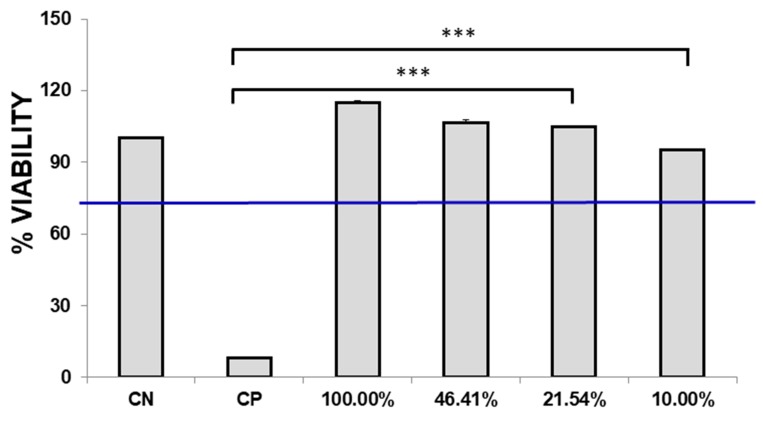
Biocompatibility test according to ISO10993-5. The graph shows the viability of L929 at all dilutions of the extract, as recommend by the ISO guidelines. CP positive control, CN negative control. *** *p*-value > 0.001.

As reported in Figure 3, specimens incubated with 100% eluates and with further dilutions displayed viability above 70%, thus confirming that BGMS10 is biocompatible according to ISO10993. Notably, specimens incubated with 100% eluates, and with dilutions of 46.41% and 21.54% resulted to be higher than the negative control: in fact, for specimen incubated with 100% of eluates, an average of 114.98 ± 0.27% was obtained. Although the difference is not statistically significant, with a *p-*value > 0.05 with respect to control samples, it is possible to hypothesize that the compounds released in the eluates exert an effect on the metabolism of BM-MSCs. In [37], Leite et al. reported similar results, obtaining an increase of the metabolism in the cell line of osteosarcoma (Saos-2) when co-cultured with bioactive glass nanoparticles. Also Nardone et al. described the biological role of strontium on MSCs proliferation [38]. However, further studies to investigate the amount of strontium released from bioactive glasses and to clarify its biological role in 3D cultures are the focus of current and future research. 

### 3.4. BM-MSCs Adhere and Colonize BGMS10 Granules

After verifying the biocompatibility of BGMS10, the aim was to predict the impact of the bioactive glass granules on bone tissue, using a 3D cellular model. Such model has been designed considering the clinical use, where biomaterials should be delivered to the operating theatre and promptly implanted. 

In [39], Detsch et al. described the osteogenic differentiation of two types of mesenchymal stem cells when seeded on 45S5 bioglass, however, before seeding, bioglasses were incubated for 2 weeks in cell culture media, and the media was changed every day. On the contrary, in this work, BGMS10 has been designed for prompt use without washes, therefore the impact of the rinse of biomaterials on cell survival was evaluated. No difference on cell adhesion and proliferation was observed on BGMS10 with or without rinses (data not shown), suggesting that the granules can be promptly implanted in vivo without performing any type of wash that could represent a bias for the subsequent in vivo studies and clinical use. 

BM-MSCs were seeded on dried BGMS10 granules, which are intrinsically 3D, at different densities to find out the proper one in terms of cells attachment, survival, and ability to colonize the biomaterial. 

It is worth noting that the combination of human BM-MSCs with the biomaterial was successfully performed on osteoinductive ceramic scaffolds [24]. In this work, we challenged the association of human BM-MSCs with bioactive glasses.

**Figure 4:**
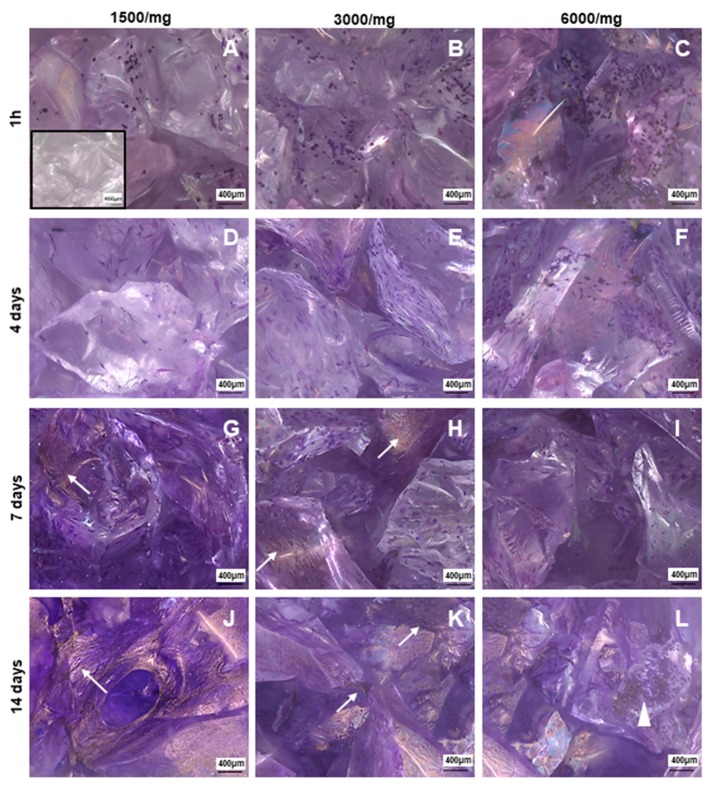
Bone marrow mesenchymal stromal/stem cells (BM-MSCs) adhered and colonized BGSM10. Representative photomicrographs of Crystal violet staining on BGSM10 at 1 hour post seeding for the three cells densities: 1500/mg (**A**), 3000/mg (**B**), and 6000/mg (**C**); at 4 days post seeding for 1500/mg (**D**), 3000/mg (**E**), and 6000/mg (**F**); at 7 days post seeding for 1500/mg (**G**), 3000/mg (**H**), and 6000/mg (**I**); and at 14 days post seeding for 1500/mg (**J**), 3000/mg (**K**), and 6000/mg (**L**). Bundles of BM-MSCs were detectable starting from 7 days of culture as show in photomicrographs (**G**,**H**) (arrow). After 14 days, tissue like structures (arrow) were rarely observed at density of 1500/mg (**J**), whilst being frequently observed (arrow) at 3000/mg (**K**). In cultures seeded with 6000/mg, cell debris was observed (arrow head), suggesting that ratio cell/mg is not suitable in static conditions. Photomicrographs were acquired with microscope AxioZoom V.16 (Zeiss) equipped with ZEN Pro software, magnification 100×. Scale bars = 400μm.

In order to identify the best amount of cells required for BGMS10 colonization, three different densities were assayed: 1500/mg, 3000/mg, 6000/mg. Then, BM-MSCs onto BGMS10 were detected by Crystal violet staining, as in a previous study [24]. In Figure 4, four time points of the 3D culture are displayed: 1 hour, 4 days, 7 days, and 14 days after seeding. For all cell densities, at 1 hour post seed, purple round shaped positive elements were observed, resembling the morphology of BM-MSCs just seeded (Figure 4A–C). At 4 days post seeding, cells spread onto the bioactive glass, showing the fibroblastoid shape and resulting homogeneously distributed on the seeded biomaterials (Figure 4D–F). Notably, at 7 days of culture (Figure 4G-I), BM-MSCs with their fibroblastoid morphology were observed, as well as a purple dark area resembled bundles of cells that colonize the biomaterials in order to generate a tissue-like structure. At 14 days post seeding (Figure 4J–L), no fibroblastoid cells were founded adherent to BGMS10, but only purple dark bundles were detectable, suggesting that BM-MSCs were able to colonize the biomaterial. 

In this last time point of the culture, it was possible to observe that BGMS10 resulted to be differently colonized in the three cell densities. In Figure 4K, referred to BGMS10 seeded with 3000/mg and kept in culture for 14 days, a greater amount of stained area was detected, compared to 1500/mg (Figure 4J) and 6000/mg (Figure 4L). We could hypothesize that in cultures seeded with 1500/mg there were a few BM-MSCs, whilst in condition of 6000/mg, detachment occurred because static conditions could not be suitable for this density. These findings addressed the selection of 3000/mg as the seeding density with the best performance in terms of attachment, survival, and proliferation. 

### 3.5. BM-MSCs Are Able to Differentiate versus Bone Lineage

Since BM-MSCs were able to adhere, spread on the material, and colonize, the osteogenic differentiation assay was performed by addition of a mix of growth factors to the expansion media. Alizarin Red staining was used to detect calcium deposits in the mineralized matrix in ex vivo bone differentiation assay, as reported in the literature [40].

**Figure 5:**
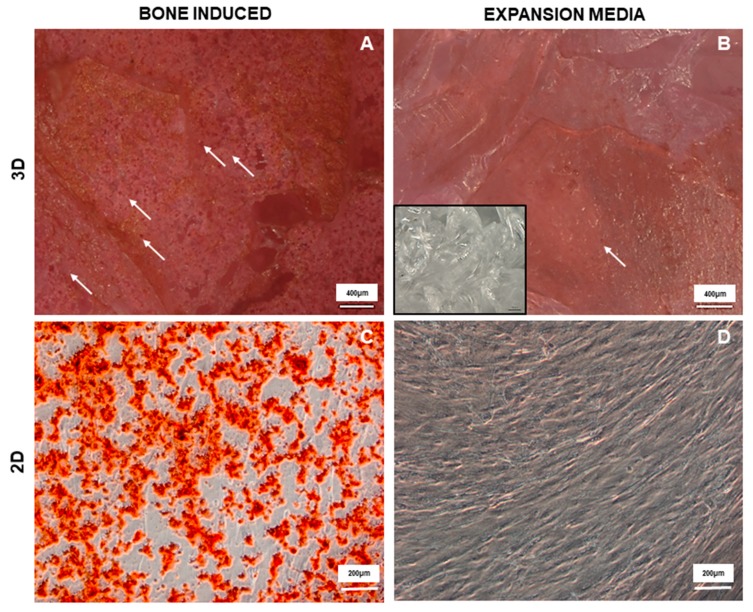
BM-MSC bone differentiated on BGSM10. Representative photomicrographs of Alizarin Red staining for induced cultures where BM-MSCs were incubated with osteogenic media (**A**); control culture where BM-MSCs were incubated with expansion media; and similarly for BM-MSCs bone differentiated in 2D standard culture, osteogenic culture (**C**) and control culture (**D**). Photomicrographs of 3D cultures were acquired with microscope AxioZoom V.16 (Zeiss) equipped with ZEN Pro software, magnification 100×. Scale bars = 400 μm (**A**-**B**); Photomicrographs of 2D cultures were acquired with microscope Axio Observer Z1 (Zeiss) equipped with ZEN Pro Software, magnification 100×. scale bars = 200 μm.

Induced cultures resulted homogeneously and completely positive to the staining, showing round bright red structure resembling calcium deposits (Figure 5A). Notably, also cultures maintained only with expansion media (Figure 5B) had resulted to the staining, highlighting the ability of the material itself to induce cells towards an osteogenic differentiation, probably due to the modification and dissolution of the material when in contact with the culture media, as already reported in literature for other bioactive glasses [41].

Ions released during the culture stimulated BM-MSCs to differentiate, even in the absence of proper growth factors, underlying the osteoinductivity of the biomaterial itself.

Also, bone differentiation on 2D cultures was performed as a control to show that BM-MSCs were able to make bone in vitro. As reported in Figure 5C, positive calcium deposits were observed in presence of induction media and not in the control culture (Figure 5D). 

These findings clearly showed that BGMS10 can support human BM-MSCs adhesion, colonization, and bone differentiation. Moreover, the results from cultures maintained only with expansion media suggested that the granules and their dissolution products are prone to influence the commitment of BM-MSCs toward osteoblast differentiation. Thus, it can be hypothesized that the ions released from the BGMS10 granules may exert a promising osteogenic effect.

## 4. Conclusions

In this work, for the first time, the novel bioactive glass BGMS10 was originally produced in form of particulate (i.e., granules with grain size between 300 and 600 μm), in order to create a suitable bone substitute for applications in orthopedics, dentistry, and in general for tissue engineering. BGMS10, which is characterized by a remarkably higher crystallization temperature (932 °C) than 45S5, maintains its amorphous nature upon thermal treatments and thus has an incredible potential in terms of bioactivity. Moreover, its composition contains strontium and magnesium, whose beneficial effects are well known. 

In this context, the results showed the great potential of BGMS10 granules. Indeed BGMS10 gave positive results in terms of SBF testing and biocompatibility according to ISO10993 standard. Moreover, a 3D cellular model that mimicked the potential clinical application of a biomaterial was here applied for the first time to a bioactive glass, with such a model designed considering the clinical use—where biomaterials have to be delivered to the operating theatre and promptly implanted—utilized human bone marrow mesenchymal stem cells (BM-MSCs). 

BM-MSCs adhered and colonized bioactive glass granules, and were able to differentiate versus bone lineage, thus highlighting the ability of BGMS10 to induce cells to osteo-differentiation. Moreover, ions released during the culture stimulated BM-MSCs to differentiate, even in the absence of proper growth factors, underlying the osteoinductivity of the biomaterial itself. In conclusion, the findings clearly showed that BGMS10 supports human BM-MSCs adhesion, colonization, and bone differentiation.
